# Phase 1 study of temozolamide (TMZ) combined with procarbazine (PCB) in patients with gliomas

**DOI:** 10.1038/sj.bjc.6601043

**Published:** 2003-07-15

**Authors:** E S Newlands, T Foster, S Zaknoen

**Affiliations:** 1 Imperial College School of Medicine, Charing Cross Hospital, Fulham Palace Road, London W6 8RF, UK; 2 Schering-Plough Research Institute K-15-3-3200, 2015 Galloping Hill Rd, Kenilworth, NJ 07033, USA

**Keywords:** temozolamide, procarbazine, glioma

## Abstract

Temozolomide (TMZ) is an oral alkylating agent with a good safety profile and proven efficacy in the treatment of malignant glioma. Procarbazine (PCB) has been used for treating gliomas for many years and here both agents were combined in the treatment. This phase I study was designed to evaluate the efficacy and safety of TMZ alone (course 1) and TMZ in combination with PCB in subsequent courses in chemotherapy-naïve patients with malignant glioma. Patients with anaplastic astrocytoma (AA), glioblastoma multiforme (GBM) and low-grade glioma were treated with TMZ 200 mg m^−2^ on days 1–5 on a 28-day cycle for course 1. Beginning with course 2, cohorts of patients received TMZ at full dose with escalating doses of PCB (50/75/100/125 mg m^−2^ days 1–5 given 1 h prior to TMZ). A total of 28 patients were enrolled with three patients each at dose level 1 and 2, 16 patients at dose level 3 and six patients at dose level 4 received 182+ cycles of treatment and were included in this analysis. In all, 16 patients had GBM, seven patients had AA, five had grade 1 or 2 glioma and the median age was 47 years. The patients had received prior surgery and radiotherapy. Responses were seen at all dose levels. Overall, there were 10 (36%) responses lasting from 2 to 17+ months. Treatment was generally well tolerated with few grade 3 or 4 toxicities, except at dose level 4, where four patients had grade 3/4 had thrombocytopaenia at this dose and several patients had moderate-to-severe lethargy. TMZ 200 mg m^−2^ and PCB 100 mg m^−2^ were well tolerated on a daily 5 × and four weekly cycle in patients with malignant glioma and clearly had antitumour activity.

Prognosis with patients with glioma, and in particular, high-grade (anaplastic astrocytoma (AA) and glioblastoma multiforme (GBM)) tumours is poor. Studies have confirmed the beneficial effect of postoperative cranial irradiation, but in most series, survival beyond 2 years is 15% or less (Walker *et al*, 1978; Stewart, 1989; Fine *et al*, 1993). The most widely used combination in treating gliomas is procarbazine, CCNU (lomustine), vincristine (PCV). This has a limited effect on survival (Levin *et al*, 1980; Sandberg-Wollheim *et al*, 1991). The most active agents identified until recently were the nitrosoureas (BCNU, CCNU, methyl-CCNU and HECNU), and have been reported to induce responses in the range of 35–55%. However, the majority of these responses were of short duration (Rodriguez and Levin, 1987; Georges *et al*, 1988; Stewart, 1989).

Temozolomide came from a synthetic programme of a number of imidazotetrazine derivatives, which exhibited broad-spectrum antitumour activity against murine models (Stevens *et al*, 1984). TMZ was selected for further clinical development in view of its good experimental antitumour activity and low toxicity in the pre-clinical screen. In addition, its antitumour activity was also schedule-dependent (Stevens *et al*, 1987). In the early clinical development of TMZ, administration of a single dose induced myelosupression but did not have any antitumour activity. However, when given on a daily 5 × schedule repeated every 4 weeks, activity against malignant melanomas and gliomas was seen (Newlands *et al*, 1992). TMZ spontaneously ring opens at physiological pH to produce the active intermediate MTIC, which methylates DNA at a number of sites. The main cytotoxic lesion induced by TMZ is probably at the *O*^6^ position of guanine (Tisdale, 1987; Baer *et al*, 1993; Wedge *et al*, 1996). This cytotoxic lesion is repaired by the DNA-repair protein O^6^-alkylguanine DNA alkyltransferase (AGT) that accepts the methyl group onto a cysteine residue and is autoinactivated. TMZ, especially administered in repeat dosing, will deplete tumour cells of AGT (D'Incalci *et al*, 1991; Mitchell and Dolan, 1993).

PCB has been used as an oral agent for many years in patients with malignant lymphomas (MOPP (mustine, vincristine, procarbazine, and prednisolone)) and in PCV-treated malignant gliomas. A number of studies in the 1990s also identified that procarbazine, a DNA-alkylating agent, depletes AGT (Schold *et al*, 1989; Souliotis *et al*, 1990; Valvanis *et al*, 1994; Russell *et al*, 1995). This study was designed to identify whether there is potentially an increase in the therapeutic index by combining PCB and TMZ in treating patients with malignant gliomas.

## PATIENTS AND METHODS

Following ethics committee approval, 28 patients with malignant gliomas were enrolled in this study and their details are shown in Table 1

**Table 1:** Clinical structure: temozolomide and procarbazine phase I

**Clinical details**	**Numbers**
Gender	
Male	17
Female	11
	
Age	
Median	47
Range	18–67
	
Diagnosis	
Glioma	
Glioma Grade 2	3
Glioma Grade 3	7
Glioma Grade 4	16
Oligodenroglioma	
Grade 2	1
Oligodenroglioma–Astrocytoma	
Grade 2	1
Total entered	28

. All had received prior surgery and radiotherapy, and none had received chemotherapy and all had progressive disease. The study design was such that TMZ alone was administered during course 1 to determine whether or not each patient's bone marrow was sensitive to TMZ at full dose. The second and subsequent courses of TMZ were combined with escalating doses of PCB (Table 2

**Table 2:** Study design: temozolomide and procarbazine phase I

	**Course**	**Period**
*Course 1*		
	Temozolomide 200 mg m^−2^ day^−1^	Days 1–5 q 28 days
*Course 2*		
Dose	Subsequent courses:	
Level 1	Procarbazine 50 mg m^−2^ day^−1^ 1 h	Days 1–5 q 28 days
	Before temozolomide 200 mg m^−2^ day^−1^	Days 1–5
Dose	Subsequent courses:	
Level 2	Procarbazine 75 mg m^−2^ day^−1^ 1 h	Days 1–5 q 28 days
	Before temozolomide 200 mg m^−2^ day^−1^	Days 1–5
Dose	Subsequent courses:	
Level 3	Procarbazine 100 mg m^−2^ day^−1^ 1 h	Days 1–5 q 28 days
	Before temozolomide 200 mg m^−2^ day^−1^	Days 1–5
Dose	Subsequent courses:	
Level 4	Procarbazine 125 mg m^−2^ day^−1^ 1 h	Days 1–5 q 28 days
	Before temozolomide 200 mg m^−2^ day^−1^	Days 1–5

). If a patient's bone marrow was sensitive to TMZ at a dose of 200 mg m^−2^ daily 5 × , this was reduced in the second course to 150 mg m^−2^ daily 5 × , and then subsequent courses combined with PCB and the reduced dose of TMZ. Patients continued on 4 weekly courses of TMZ at the same dose of PCB until disease progression or evidence of major toxicity.

Responses were assessed clinically and radiologically before and after each course of treatment. Patients with gliomas had to be taking a stable dose of corticosteroid for at least 2 weeks before study entry and the baseline CT or MRI scan. Radiological shrinkage alone was not considered an acceptable measure of response in brain tumours, as there is often difficulty in forming clear margins on a CT or MRI abnormality, which includes additional areas of necrosis, oedema, and vascularity. In some cases, it may be possible to measure a clearcut reduction of 50% by CT or MRI, but in most cases it is only possible to determine a reduction in enhancement and mass effect. An objective response (OR) was defined as one that requires improvement in the Medical Research Council neurological status (Table 3

**Table 3:** Medical Research Council Neurological Status

0	No neurological deficit
1	Some neurological deficit, but function adequate for useful work
2	Neurological deficit causing moderate functional impairment (e.g. being able to move limb(s)) only with difficulty, moderate dysphasia, moderate paresis, and some visual disturbances (e.g. visual field defect)
3	Neurological deficit causing major functional impairment for example inability to move limbs, gross speech, or visual disturbance
4	No useful function; inability to make conscious responses

) by one grade as well as a clear-cut reduction in mass effect on CT or MRI assessment with a minimum duration of 4 weeks. There should also be no deterioration in any other neurological symptom or sign and no development of new neurological deficits. Responses had to be documented by two observations at least 4 weeks apart (Brock *et al*, 1998).

## RESULTS

In general, the combination of TMZ and PCB was well tolerated. Dose level 1 (PCB 50 mg m^−2^ per day given 1 h prior to TMZ) from course 2 onwards was well tolerated without any major side effects, as was dose level 2 (PCB 75 mg m^−2^ daily × 5). The only significant toxicity at these two dose levels was lymphocytopenia (Table 4

**Table 4:** Haematological toxicities. Common toxicity criteria grades 3 and 4

	**Dose level 1**	**Dose level 2**	**Dose level 3**	**Dose level 4**
	**3 patients**	**3 patients**	**16 patients**	**6 patients**
Anaemia	0	0	0	0
Leucopenia	0	0	1^a^	0
Lymphocytopenia	1	3	2^b^	1^a^
Neutropenia	0	0	1^a^	0
Thrombocytopenia	0	0	4^c^	4

a Temozolomide alone.

b Temozolomide alone, one patient.

c Temozolomide alone, three patients.

). At dose level 3 (PCB 100 mg m^−2^ at daily × 5), two patients had falls in their white counts and four patients had thrombocytopenia all on TMZ alone. One patient had thrombocytopenia from the combination of TMZ and PCB. At dose level 4, toxicity was seen and four patients had thrombocytopenia and several patients had moderate-to-severe lethargy and malaise. The recommended dose for future studies is dose level 3. The other toxicities that were seen were skin rashes (two patients) and one patient had hepatitis A infection (Table 5

**Table 5:** Other toxicities. Common toxicity criteria grades 3 and 4

	**Dose level 1**	**Dose level 2**	**Dose level 3**	**Dose level 4**
	**3 patients**	**3 patients**	**16 patients**	**6 patients**
Pain/headaches	0	0	1	1
Stomatitis	0	0	0	0
Infection	0	0	1^a^	0
Skin rash	0	0	2	0
Raised (ALT) transaminases	0	1	1^b^	1^a^
Raised bilirubin	0	0	1^b^	0

a Temozolomide alone.

b Hepatitis A infection, one patient.

).

The responses by tumour grade are shown in Table 6

**Table 6:** Response to histological grade

**Tumour grade**	**Response %**	**Stable disease**	**Progressive disease**	**Total patient no.**
2	1 (20)	1	3	5
3 (AA)	3 (50)	1	2	6
4 (GBM)	6 (35)	0	11	17
TOTAL (%)	10 (36)	2 (7)	16 (57)	28

AA=anaplastic astrocytoma, GBM=glioblastoma multiforme.

. Responses were seen in all types of glioma, with an overall response rate of 36%. Table 7

**Table 7:** Responses by dose of procarbazine

**Dose level**	**Response (%)**	**Stable disease**	**Progressive disease**	**Total patient no.**
Procarbazine 50 mg m^−2^ × 5	1 (33)	0	2	3
Procarbazine 75 mg m^−2^ × 5	1 (33)	0	2	3
Procarbazine 100 mg m^−2^ × 5	5 (31)	1	10	16
Procarbazine 125 mg m^−2^ × 5	3 (50)	1	2	6
Total (%)	10 (36)	2 (7)	16 (57)	28

shows the response by PCB dose and responses were seen at all dose levels. Table 8

**Table 8:** Response duration

**Procarbazine dose level**	**Months**
1	17+
2	11
3	6+, 7+, 8+, 13+, 14+
4	2, 5+, 6+

shows the duration of responses to 1 September 2001, the duration of responses lasting from 2 to 17+ months. Figure 1

**Figure 1:**
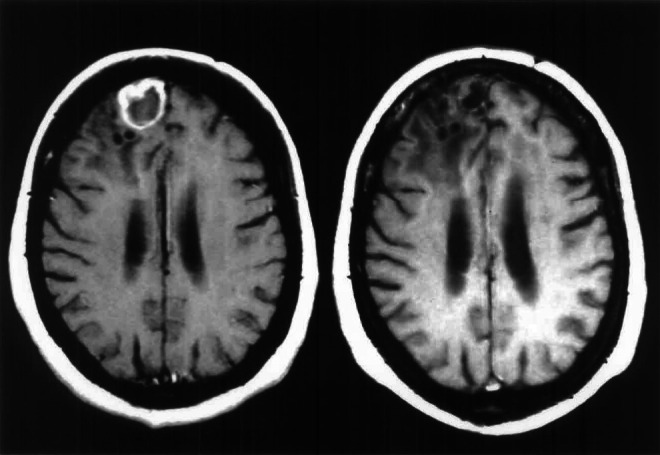
Glioma grade II patient transforming to GBM. This MRI shows a patient with grade II glioma, which had transformed to GBM. This is an ongoing response to TMZ and PCB at dose level 3 at 14+ months.

shows a response in a patient receiving treatment at dose level 3.

## DISCUSSION

Using the criteria of response, we have previously published responses in patients with high-grade gliomas (AA and GBM) to TMZ that are well documented and approximately one in four patients achieves an objective response with clearcut neurological improvement and reduction in the area of enhancement on their MRI scan (Brock *et al*, 1998). A further 25% have disease stabilisation in terms of their MRI scans and some neurological improvement when TMZ is given in the dose of 200 mg m^−2^ days 1–5 schedule repeated at 4 weekly intervals. In the randomised study of TMZ against PCB, the response rate to PCB was lower, the duration of those responses shorter, and the quality of life poorer. However, PCB clearly had modest activity against high-grade gliomas (Yung *et al*, 2000).

In this study, the combination of TMZ and PCB was generally well tolerated at doses up to dose level 3. At this dose level, there is little difference in terms of side effects between TMZ alone and the combination with PCB. We had intended to extend this cohort to 30 or 40 patients to identify whether or not the combination was superior to TMZ on its own. However, the supply of PCB was interrupted and the study had to be suspended when a total of 16 patients had been entered at dose level 3. Clearly, these results on a small number of patients are preliminary. The clinical impression is that the combination probably is a bit more active than TMZ on its own, and it may be that some of the patients who would otherwise have been classified as having stable disease would have moved into the responding group, suggesting a greater antitumour activity when the two agents are given together.

## CONCLUSION

TMZ and PCB, when combined at the recommended dose levels, are a reasonably effective and well-tolerated combination in treating patients with relapsed gliomas and tumour activity is seen in low-grade gliomas, AA, and GBM.
